# Outcome of hepatic resection for HCC in ideal and non-ideal candidates

**DOI:** 10.1097/HC9.0000000000000772

**Published:** 2025-07-29

**Authors:** Lorenzo Lani, Laura Bucci, Valentina Santi, Benedetta Stefanini, Bernardo Stefanini, Angelo Sangiovanni, Sara Grasselli, Giorgia Ghittoni, Carlo Saitta, Filomena Morisco, Giuseppe Cabibbo, Fabio Marra, Gianpaolo Vidili, Maurizia Rossana Brunetto, Francesco Giuseppe Foschi, Mariella Di Marco, Gianluca Svegliati-Baroni, Filippo Pelizzaro, Francesco Azzaroli, Francesca Romana Ponziani, Andrea Martini, David Sacerdoti, Andrea Mega, Sara Boninsegna, Edoardo G. Giannini, Donatella Magalotti, Rodolfo Sacco, Gerardo Nardone, Paolo Caraceni, Alessandro Vitale, Franco Trevisani, and the Italian Liver Cancer group

**Affiliations:** 1 Unit of Semeiotics, Liver and Alcohol-related Diseases, IRCCS Azienda Ospedaliero-Universitaria di Bologna, Bologna, Italy; 2 Department of Medical and Surgical Sciences, University of Bologna, Bologna, Italy; 3 Division of Internal Medicine, Hepatobiliary and Immunoallergic Diseases, IRCCS Azienda Ospedaliero-Universitaria di Bologna, Bologna, Italy; 4 Division of Gastroenterology and Hepatology, Fondazione IRCCS Ca’ Granda Ospedale Maggiore Policlinico Milan, Italy; 5 Department of Medicine and Surgery, Infectious Diseases and Hepatology Unit, University of Parma and Azienda Ospedaliero-Universitaria of Parma, Parma, Italy; 6 Gastroenterology Unit, Belcolle Hospital, Viterbo, Italy; 7 Department of Clinical and Experimental Medicine, Clinical and Molecular Hepatology Unit, University of Messina, Messina, Italy; 8 Department of Clinical Medicine and Surgery, Diseases of the Liver and Biliary System Unit, University of Naples “Federico II,” Naples, Italy; 9 Section of Gastroenterology and Hepatology, Department of Health Promotion, Mother and Child Care, Internal Medicine and Medical Specialties, PROMISE, Gastroenterology and Hepatology Unit, University of Palermo, Palermo, Italy; 10 Department of Experimental and Clinical Medicine, Internal Medicine and Hepatology Unit, University of Florence, Florence, Italy; 11 Department of Medicine, Surgery and Pharmacy, Azienda Ospedaliero-Universitaria of Sassari, Sassari, Italy; 12 Department of Clinical and Experimental Medicine, Hepatology and Liver Physiopathology Laboratory, University Hospital of Pisa, Pisa, Italy; 13 Department of Internal Medicine, Ospedale per gli Infermi di Faenza, Faenza, Italy; 14 Medicina Ospedale Bolognini Seriate, ASST Bergamo Est, Seriate, Italy; 15 Liver Injury and Transplant Unit, Polytechnic University of Marche, Ancona, Italy; 17 Division of Gastroenterology, IRCCS Azienda Ospedaliero-Universitaria di Bologna, Bologna, Italy; 16 Department of Surgery, Oncology and Gastroenterology, Gastroenterology Unit, University of Padua, Padua, Italy; 18 Liver Unit, CEMAD—Centro Malattie dell’Apparato Digerente, Medicina Interna e Gastroenterologia, Università Cattolica del Sacro Cuore, Fondazione Policlinico Universitario Gemelli IRCCS, Rome, Italy; 19U.O.C Clinica Medica 5, Unit of Internal Medicine and Hepatology, Department of Medicine, Azienda Ospedale Università Padova, Padova, Italy; 20 Liver Unit, Department of Medicine, University of Verona, Azienda Ospedaliera Universitaria Integrata of Verona, Verona, Italy; 21 Gastroenterology Unit, Bolzano Regional Hospital, Bolzano, Italy; 22 Gastroenterology Unit, Ospedale Sacro Cuore Don Calabria, Negrar, Italy; 23 Gastroenterology Unit, Department of Internal Medicine, University of Genoa, Genoa, Italy; 24 IRCCS Ospedale Policlinico San Martino, Genoa, Italy; 25 Division of Internal Medicine, Neurovascular and Hepatometabolic Diseases, IRCCS Azienda Ospedaliero-Universitaria di Bologna, Bologna, Italy; 26 Gastroenterology and Digestive Endoscopy Unit, Foggia University Hospital, Foggia, Italy; 27 Department of Clinical Medicine and Surgery, Hepato-Gastroenterology Unit, University of Naples “Federico II,” Naples, Italy; 28 Italian Liver Cancer Association, Bologna, Italy

**Keywords:** clinically significant portal hypertension, hepatic resection, HCC, hyperbilirubinemia, liver resection

## Abstract

**Background:**

The Barcelona Clinic Liver Cancer staging system considers, among patients with HCC, “ideal candidates” (ICs) for hepatic resection (HR) those with a single lesion, normal bilirubin, and without clinically significant portal hypertension (CSPH). We compared the outcome of HR between ICs and non-ICs.

**Methods:**

Retrospective analysis was conducted on Child–Pugh A patients. CSPH was defined by the presence of gastroesophageal varices and/or platelet count <100,000/mm^3^. Hyperbilirubinemia was accepted up to 2 mg/dL. The selected 1057 patients were distributed in 3 calendar periods (2000–2022).

**Results:**

In all calendar periods, non-ICs were more prevalent than ICs. Among non-ICs, the proportion of patients with isolated CSPH did not change over time (from 22.6% to 30.3%; *p*=0.359), while patients with multinodular HCC (mHCC) increased (from 35.5% to 50.2%; *p*=0.042). Patients with hyperbilirubinemia decreased (from 20.4% to 10.1%; *p*=0.036), likewise those with hyperbilirubinemia+CSPH (from 21.5% to 9.4%; *p*=0.005). Over a median follow-up of 41.0 months, median overall survival was higher in ICs compared to non-ICs (104.9 vs. 75.3 months; *p*<0.001). However, compared to ICs, median overall survival did not differ in patients with isolated CSPH (93.1 mo; *p*=0.432) or isolated hyperbilirubinemia (86.0 mo; *p*=0.356), while it was lower in those with hyperbilirubinemia+CSPH (60.0 mo; *p*<0.001) or mHCC (61.9 mo; *p*<0.001). Compared to ICs, only hyperbilirubinemia+CSPH patients showed a higher perioperative mortality.

**Conclusions:**

In real-world practice, among resected patients, the proportion of non-ICs has remained constantly higher than that of non-ICs since 2000. HR can be offered to Child–Pugh A patients with CSPH or modest hyperbilirubinemia without compromising its outcome. For patients with 2 of these features or mHCC, which generate a poorer prognosis, studies comparing HR versus non-surgical therapies are warranted.

## INTRODUCTION

Hepatic resection (HR) is a mainstay of the treatment of HCC and, in terms of survival benefit, ranks second after liver transplantation.^1^ However, as most HCCs arise in a cirrhotic liver, its use is limited by the presence of deteriorated liver function and/or clinically significant portal hypertension (CSPH).

The 2024 version of European guidelines states that all patients with HCC free from distant metastasis should be considered for HR by a Multidisciplinary Tumor Board (MTB), with an indication based on a multiparametric assessment of liver function, portal hypertension (PH), and extent of hepatectomy.^2^ Nevertheless, the 2022 updated Barcelona Clinic Liver Cancer staging system considers as “ideal candidates” (ICs) for HR patients with a solitary HCC, preserved liver function (Child–Pugh A) with normal bilirubin level, no evidence of CSPH, excellent performance status (PS), and without macrovascular invasion and extrahepatic cancer spread on imaging.^3^ This distinction, which has been retained by the last version of the American guidelines,^4^ derives from results of a study including 77 patients resected in the late ‘90s in Barcelona.^5^ This approach selects patients in whom HR is expected to provide the best outcome, and tends to exclude from surgery “non-ideal candidates” (non-ICs) in whom HR potentially outperforms non-surgical therapies. Actually, real-world data from expert centers indicate that many patients undergoing HR are non-ICs due to the presence/co-presence of CSPH, hyperbilirubinemia, or multinodular HCC (mHCC)^6^^–^^10^ and, in large cohorts of patients with mHCC matched with propensity score (PSM), HR led to a better long-term survival compared to radiofrequency ablation or transarterial chemoembolization (TACE).^11^^–^^19^ Using PSM, a huge study showed that HR outperformed non-surgical treatments in patients with intrahepatic macrovascular invasion, although at the time of this study sorafenib had not still become a standard treatment.^20^ Accordingly, more recent Western studies report a superiority of HR over systemic therapy in selected patients with locally advanced HCC.^21^^,^^22^ Lastly, the treatment upward (toward potentially more effective approaches) with respect to the Barcelona Clinic Liver Cancer (BCLC) indications would improve patient prognosis, particularly in those with intermediate and advanced stage HCC.^23^^–^^25^

It is also pertinent to consider that, since ICs were defined, minimally invasive surgery based on laparoscopic or robotic approaches, advancements in post-operative management, and the positive effect of antiviral therapy on liver function have extended the feasibility of HR without compromising the outcome. Therefore, both Korean and Italian guidelines for the management of patients with HCC allow minor resections in selected patients with CSPH or with 2–3 nodules.^26^^,^^27^ Japanese and Asian guidelines adopt even less stringent selection criteria, recommending surgery for multiple tumors and for tumors invading the intrahepatic portal branches.^28^^,^^29^

Considering the substantial variability across different centers and regions regarding the use of HR for the treatment of HCC, this study aimed to provide further insights into this still hot topic by answering the following questions:

- Have the proportions of ICs and non-ICs cases among resected patients changed over time (period 2000–2022)?
- Has the composition of the non-IC population changed over time?
- Can the selection criteria for HR be expanded without detrimental consequences on perioperative and long-term outcomes compared to ICs, and how?

## METHODS

### Patients

Data were extracted from the ITA.LI.CA database, which currently includes 10,907 patients with HCC consecutively recruited from January 1986 to December 2022 by 26 medical institutions in Italy. Data were collected prospectively and updated every 2 years. ITA.LI.CA centers collect data at the time of HCC diagnosis (baseline) and during follow-up. The management of the ITA.LI.CA database conforms to the current Italian legislation on privacy. According to Italian law, patient consent is not required for retrospective data analysis. Nonetheless, all patients provided written informed consent to undergo each diagnostic and therapeutic procedure and to have their clinical data recorded anonymously in the ITA.LI.CA database. The use of this database for scientific research was approved by the Institutional Review Board Comitato Etico Area Vasta Emilia Centro (CE-AVEC), Emilia-Romagna region (approval number 99/2012/O/Oss), and the study was conducted following the ethical guidelines of the Declaration of Helsinki and Istanbul.

**FIGURE 1:**
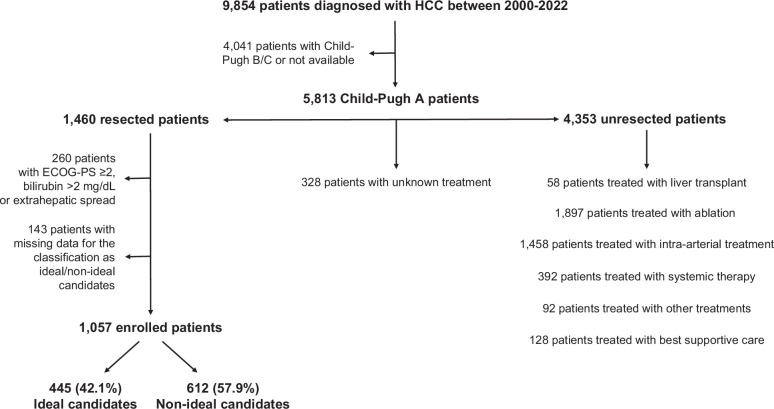
Flowchart describing the construction of the final analytic dataset of Child–Pugh A patients with HCC treated with hepatic resection or other treatments. Abbreviation: ECOG-PS, Eastern Cooperative Oncology Group-Performance Status.

In the selected calendar period (from 1 January 2000 to 31 December 2022), 5813 consecutive Child–Pugh A patients with HCC were identified. Of these, 1460 patients underwent hepatic resection, and 4353 received other treatments. Among resected patients, 260 cases were excluded because of the presence of Eastern Cooperative Oncology Group-Performance Status (ECOG-PS) ≥2, bilirubin >2mg/dL, or extrahepatic spread, and 143 patients were excluded because of missing data that precluded an appropriate classification. The final sample included 1057 patients (Figure 1).

According to tumor burden (solitary vs. multinodular), serum bilirubin level (≤1.1 mg/dL vs. 1.2–2.0 mg/dL) and presence/absence of CSPH, patients were classified as ICs (without any adverse feature), or non-ICs (with at least one adverse feature). Non-ICs were further subdivided as follows:

- Patients with isolated hyperbilirubinemia (bilirubin 1.2–2.0 mg/dL).
- Patients with isolated CSPH (defined as the presence of gastroesophageal varices and/or platelet count <100,000/µL) without ascites.
- Patients with hyperbilirubinemia+CSPH.
- Patients with mHCC (monolobar in 54%, bilobar in 39%, and not reported in 7% of cases).

To assess the evolution of indications for HR during the study period, patients were also subdivided into 3 calendar periods according to the date of HR: T1=2000–2008, T2=2009–2015, and T3=2016–2022.

The following variables were analyzed: age, sex, presence of CSPH, serum bilirubin, etiology of liver disease (viral/non-viral), antiviral therapy (yes/no), ECOG-PS (categorized as 0/1), MELD score, tumor number (solitary/multifocal), size of the greatest lesion, presence of intrahepatic macrovascular invasion (MVI), serum alpha-fetoprotein (AFP), extension of HR [categorized as ≤3 segments vs. >3 segments or also as right (segments 5–8) or left (segments 1, 2–4) hepatic lobectomies/others], previous HCC treatment, time to HCC relapse (early: ≤2 y, late: >2 y from HR), downstream treatments, median follow-up, patient survival, and causes of death. Overall survival (OS) was calculated from the date of HR to that of death, lost to follow-up, or end of the study, whichever occurred first. All these variables were available in >80% of the cases.

If a recent upper endoscopy was considered mandatory, esophageal varices were graded and managed according to the Baveno guidelines available at the time of HCC diagnosis.

### Diagnosis and staging

The diagnosis of HCC was based on typical features of imaging techniques [dynamic CT, MRI, and contrast-enhanced ultrasonography (CEUS)] and/or histological findings, according to the European and American guidelines available at the time of patient recruitment.

Cancer burden was assessed using liver CT and/or MRI. Further investigations aimed at detecting extrahepatic involvement were systematically performed in patients with advanced HCC or those eligible for liver transplant (LT). These explorations were also repeated when clinically indicated.

HCC was staged according to the ITA.LI.CA staging system.^30^

### Statistical analysis

Continuous variables are expressed as median and 25th–75th percentiles, and discrete variables as absolute and relative frequencies. Kruskal–Wallis and Mann–Whitney *U* tests were used to compare continuous variables among the 3 periods, as appropriate. The *X* ^2^ test, or the Fisher exact test, was used to compare discrete variables. OS was calculated according to the Kaplan–Meier method and was compared using the Log-rank test. It was reported as median and 95% CI. Patients undergoing LT were censored at the time of LT. Survival rates at 3 and 6 months, and at 1, 3, and 5 years were reported.

Time to next treatment (TTNT) was calculated as the time elapsed from the index HR to the first downstream treatment for disease recurrence.

A 2-tailed *p* value <0.05 was considered statistically significant. All statistical analyses were performed with SPSS v28.0 (Apache Software Foundation).

## RESULTS

### Demographic and clinical characteristics

**FIGURE 2:**
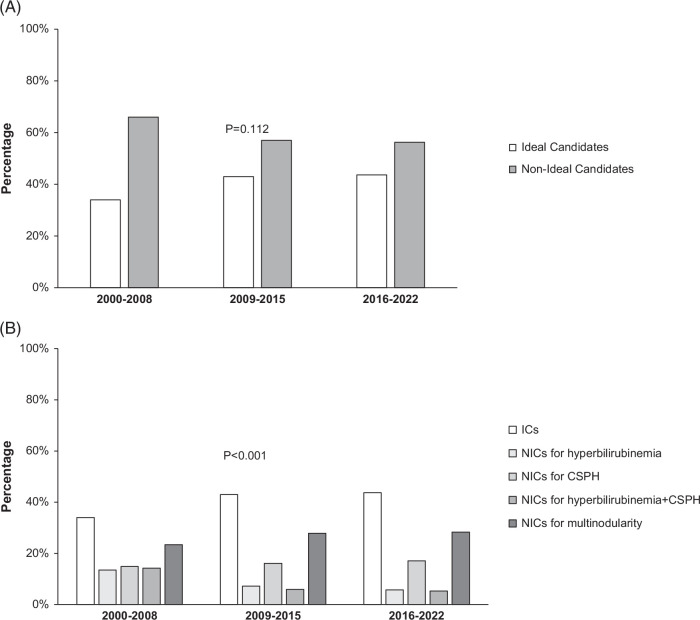
Proportion of ideal and non-ideal candidates across the study periods (A); overtime distribution of non-ideal candidates according to the cause preventing the inclusion into the ideal group (B). Abbreviations: ICs, ideal candidates; NICs, non-ideal candidates; CSPH, clinically significant portal hypertension.

Considering the entire study period, most patients were non-ICs [612 (57.9%) vs. ICs 445 (42.1%)]. The proportion of ICs tended to increase, although not significantly, over time: T1: 48 (34.0%); T2: 190 (43.0%); T3: 207 (43.7%) (*p*=0.112) (Figure 2A).

Among the 244 patients with CSPH (alone or with hyperbilirubinemia), this feature was revealed by the presence of esophageal varices in 171 (70.0%), while in the 19 patients lacking endoscopy and in the 54 without varices, CSPH diagnosis was based on platelet count <100,000/mm^3^. Among non-ICs, the overtime analysis showed that the proportion of patients with isolated CSPH did not change over time (from 22.6% to 30.3%; *p*=0.359), while that of patients with multinodular HCC (mHCC) increased (from 35.5% to 50.2%; *p*=0.042). Patients with hyperbilirubinemia (from 20.4% to 10.1%; *p*=0.036) and hyperbilirubinemia+CSPH (from 21.5% to 9.4%; *p*=0.005) decreased over time (Figure 2B).

Demographics and clinical data of patients are summarized in Table 1. Age, sex, and etiology of liver disease were similar in ICs and non-ICs, who instead significantly differed for some baseline features indicating a better liver function in ICs.

**TABLE 1:** Demographic and clinical characteristics of patients

	Ideal candidates N=445	Non-ideal candidates N=612				
		Hyperbilirubinemia N=78	CSPH N=173	Hyperbilirubinemia+CSPH N=71	mHCC N=290	*p*
Age (y)						0.076
** **N (%)	445 (100)	78 (100)	173 (100)	71 (100)	290 (100)	
Median (25–75)	69 (60–75)	69 (61–75)	67 (61–73)	65 (57–72)	68 (61–73)	
Sex						0.14
N (%)	445 (100)	78 (100)	173 (100)	71 (100)	290 (100)	
M	341 (76.6)	67 (85.9)	129 (74.6)	53 (74.6)	236 (81.4)	
F	104 (22.4)	11 (14.1)	44 (25.4)	18 (25.4)	54 (18.6)	
Type of HCC diagnosis						**0.025**
N (%)	425 (95.5)	74 (94.9)	168 (97.1)	65 (91.5)	265 (91.4)	
Surveillance	240 (56.5)	38 (51.4)	117 (69.6)	45 (69.2)	148 (55.8)	
Incidental	144 (33.9)	26 (35.1)	40 (23.8)	18 (27.7)	95 (35.8)	
Symptomatic	41 (9.6)	10 (13.5)	11 (6.5)	2 (3.1)	22 (8.3)	
Etiology						0.121
N (%)	417 (93.7)	70 (89.7)	165 (95.4)	66 (93.0)	280 (96.6)	
Viral	280 (67.3)	41 (58.6)	120 (72.7)	42 (63.6)	171 (65.9)	
Non-viral	136 (32.7)	29 (41.4)	45 (27.3)	24 (36.4)	104 (37.8)	
Anti-viral therapy ^a^						**<0.001**
N, (%)	280 (100)	41 (100)	120 (100)	42 (100)	171 (100)	
Yes	162 (67.9)	20 (48.8)	81 (67.5)	27 (64.3)	81 (47.4)	
Bilirubin, mg/dL						**<0.001**
N (%)	445 (100)	78 (100)	173 (100)	71 (100)	290 (100)	
Median (25–75)	0.7 (0.5–0.9)	1.3 (1.2–1.5)	0.8 (0.6–0.9)	1.5 (1.2–1.6)	0.8 (0.6–1.1)	
CSPH						**<0.001**
N (%)	445 (100)	78 (100)	173 (100)	71 (100)	254 (87.6)	
Yes	0 (0)	0 (0)	173 (100)	71 (100)	102 (40.2)	
No	445 (100)	78 (100)	0 (0)	0 (0)	152 (59.8)	
Platelet count, 100×10^3^/mm^3^						**<0.001**
N, (%)	445 (100)	78 (100)	169 (97.1)	70 (98.6)	284 (95.9)	
Median (25–75)	194 (150–247)	184 (137–229)	94 (75–136)	95 (72–122)	147 (108–204)	
Gastric or esophageal varices						**<0.001**
N (%)	439 (98.7)	78 (100)	158 (91.3)	67 (94.4)	254 (87.6)	
Yes	0 (0.0)	0 (0.0)	116 (73.4)	55 (82.1)	71 (28.0)	
Gross tumor pathology						**<0.001**
N (%)	445 (100)	78 (100)	173 (100)	71 (100)	290 (100)	
Single	445 (100)	78 (100)	173 (100)	71 (100)	0 (0.0)	
Multifocal	0 (0.0)	0 (0.0)	0 (0.0)	0 (0.0)	290 (100)	
Number of nodules in multifocal HCC						ND
N (%)	—	—	—	—	290 (100)	
2–3					195 (67.2)	
>3					42(14.5)	
Not reported					53 (18.3)	
Size (main nodule in multifocal HCC), cm						**<0.001**
N (%)	438 (98.4)	77 (98.7)	166 (96.0)	70 (98.6)	277 (95.5)	
Median (25–75)	3.5 (2.2–5.0)	4.0 (2.5–6.0)	2.6 (2.0–4.0)	2.7 (1.8–4.0)	3.0 (2.0–4.7)	
Size (main nodule in multifocal) HCC, cm						**<0.001**
N (%)	438 (98.4)	77 (98.7)	166 (96.0)	70 (98.6)	277 (95.5)	
≤3 cm	191 (43.6)	26 (33.8)	100 (60.2)	42 (60.0)	141 (50.9)	
>3 cm	247 (56.4)	51 (66.2)	66 (39.8)	28 (40.0)	136 (49.1)	
ECOG-PS						**0.005**
N (%)	444 (99.8)	78 (100)	172 (99.4)	71 (100)	290 (100)	
0	420 (94.6)	70 (89.7)	158 (91.9)	62 (87.3)	252 (86.7)	
1	24 (5.4)	8 (10.3)	14 (8.1)	9 (12.7)	38 (13.1)	
MELD score						**<0.001**
N, (%)	438 (98.4)	90 (97.8)	171 (98.3)	70 (98.6)	291 (98.3)	
Median (Q1–Q3)	7 (7–8)	9 (8–10)	8 (7–8)	10 (9–11)	8 (7–9)	
Naive patients						**<0.001**
N (%)	445 (100)	78 (100)	173 (100)	71 (100)	290 (100)	
Yes	398 (89.4)	72 (92.3)	147 (85.0)	63 (88.7)	228 (79.6)	
No	47 (10.6)	6 (87.7)	26 (15.0)	8 (11.3)	62 (21.4)	
Alpha-fetoprotein						0.213
N (%)	374 (84.0)	64 (82.1)	158 (91.3)	66 (93.0)	244 (84.1)	
≤400 ng/ml	332 (89.0)	60 (93.8)	149 (94.9)	60 (90.9)	225 (92.2)	
>400 ng/ml	41 (11.0)	4 (6.2)	8 (5.1)	6 (9.1)	19 (7.8)	
Extension of HR						**<0.001**
N (%)	395 (88.8)	56 (71.8)	157 (90.8)	60 (84.5)	267 (92.1)	
Major resections (> 3 segments)	4 (1.0)	1 (1.8)	1 (0.6)	0 (0.0)	30 (11.2)	
Minor resections (≤3 segments)	391 (99.0)	55 (98.2)	156 (99.4)	60 (100.0)	237 (88.8)	
N (%)	395 (88.8)	56 (71.8)	157 (90.8)	60 (84.5)	267 (92.1)	
Major resections (right or left hepatic lobectomy)	4 (1.0)	1 (1.8)	1 (0.6)	0 (0.0)	8 (3.0)	
Minor resections (others)	391 (99.0)	55 (98.2)	156 (99.4)	60 (100.0)	259 (97.0)	
Intrahepatic MVI						**0.018**
N (%)	445 (100)	78 (100)	173 (100)	71 (100)	290 (100)	
Yes	5 (1.1)	3 (3.8)	9 (5.2)	3 (4.2)	15 (5.2)	
Recurrence						**<0.001**
N (%)	445 (100)	78 (100)	173 (100)	71 (100)	290 (100)	
Yes	187 (42.4)	24 (30.8)	91 (52.9)	30 (42.3)	173 (60.1)	
TTNT, months						**0.012**
N (%)	187 (100)	24 (100)	91 (100)	30 (100)	173 (100)	
Median (Q1–Q3)	17 (10–36)	23 (11–41)	22 (13–34)	16 (9–36)	14 (8–27)	
ITA.LI.CA stage						**<0.001**
N (%)	366 (75.5)	63 (80.8)	150 (86.7)	65 (91.5)	195 (67.2)	
0	86 (23.5)	14 (22.2)	57 (38.0)	21 (32.3)	35 (17.9)	
A	200 (54.6)	32 (50.8)	77 (51.3)	33 (50.8)	47 (24.1)	
B1	80 (21.9)	17 (27.0)	16 (10.7)	11 (16.9)	51 (26.2)	
B2	0 (0.0)	0 (0.0)	0 (0.0)	0 (0.0)	56 (28.7)	
B3	0 (0.0)	0 (0.0)	0 (0.0)	0 (0.0)	6 (3.1)	
Causes of death						**0.984**
N (%)	146 (100)	34 (100)	65 (100)	41 (100)	139 (100)	
Tumor progression	75 (51.4)	16 (47.1)	28 (43.1)	17 (41.5)	66 (47.5)	
Liver failure	16 (11.0)	5 (14.7)	9 (13.8)	11 (26.8)^b^	18 (12.9)	
Hemorrhage	4 (2.7)	0 (0.0)	4 (6.2)	1 (2.4)	4 (2.9)	
Renal failure	0 (0.0)	0 (0.0)	1 (1.5)	0 (0.0)	1 (0.7)	
Infections	3 (2.1)	1 (2.9)	3 (4.6)	1 (2.4)	3 (2.2)	
Pulmonary embolism	2 (1.4)	0 (0.0)	0 (0.0)	0 (0.0)	1 (0.7)	
CVD	4 (2.7)	1 (2.9)	4 (6.2)	1 (2.4)	7 (5.0)	
Extrahepatic cancers	8 (5.5)	2 (5.9)	2 (3.1)	0 (0.0)	6 (4.3)	
Others	3 (2.1)	1 (2.9)	2 (3.1)	0 (0.0)	5 (3.6)	
Not reported	31 (21.2)	8 (23.5)	12 (18.5)	10 (24.4)	28 (20.1)	

^a^ Percentage refers to patients with viral etiology.

^b^ *p*=0.010 compared to ideal candidates.

Abbreviations: CSPH, clinically significant portal hypertension; CVD, cardiovascular disease; ECOG-PS, Eastern Cooperative Oncology Group-Performance Status; HR, hepatic resection; ITA.LI.CA, Italian Liver Cancer; MVI, macrovascular invasion; TTNT, time to next treatment.

MVI was uncommon, occurring in 1.1% of ICs, 3.8% of hyperbilirubinemic patients, 5.2% of CSPH patients, 4.2% of those with hyperbilirubinemia+CSPH, and 5.2% of those with mHCC (*p*=0.018). MVI prevalence did not change over time: 2.8% in T1, 4.5% in T2, and 2.3% in T3.

Among non-ICs with mHCC, 67.3% had 2–3 nodules and 14.5% >3 nodules; in 53 (18.2%) patients, this information was missing.

Major resections (>3 hepatic segments) were performed only in 36 patients (3.4%), with a significantly higher frequency in non-ICs with mHCC compared to the other groups.

### Tumor recurrence, TTNT (Table 1), and downstream treatments (Table 2)

**TABLE 2:** Downstream treatments adopted for tumor recurrence

Post-resection treatment	Ideal candidates N=187	Non-ideal candidates N=318	*p*
Liver transplant, N (%)	18 (9.6)	34 (10.7)	0.703
Resection, N (%)	31 (16.6)	39 (12.3)	0.176
Ablation, N (%)	70 (37.4)	124 (39.0)	0.728
IAT, N (%)	81 (43.3)	140 (44.0)	0.877
Systemic therapy, N (%)	72 (38.5)	114 (35.8)	0.551
Others, N (%)	32 (17.1)	59 (18.6)	0.684

Abbreviation: IAT, intra-arterial therapies.

During the follow-up, 505 patients (48.1%) had tumor recurrence [datum not available in 7 cases (0.7%)]. HCC recurrence was less frequent in ICs than in non-ICs (42.4% vs. 52.2%, *p*=0.002]. The highest recurrence rate occurred in patients with mHCC (60.1%), followed by those with CSPH (52.9%), hyperbilirubinemia+CSPH (42.3%), and hyperbilirubinemia (30.8%) (*p*<0.001).

Median (25th–75th) TTNT was 17.6 months (10.0–36.0) for ICs, 22.5 months (7.0–40.9) for patients with hyperbilirubinemia, 22.0 months (12.0–5.5) for patients with CSPH, 16.1 months (5.6–35.9) for those with hyperbilirubinemia+CSPH, and 13.5 months (8.0–25.8) for those with mHCC (*p*=0.012).

An early (≤2 y) recurrence occurred in 61.5% of ICs and in 67.6% of non-ICs (*p*=0.163). Among non-ICs, it was more frequent in patients with mHCC (74.6%), followed by those with hyperbilirubinemia (66.7%), with hyperbilirubinemia+CSPH (63.3%), and with CSPH (56.0%).

The distribution of downstream treatments for disease recurrence did not differ between ICs and non-ICs. About 10% of patients underwent LT in both groups, while re-resection accounted for 16.6% and 12.3% of cases, respectively. The most common treatment was intra-arterial therapy (IAT), and systemic therapy was utilized in more than one-third of cases in both groups.

### Causes of death, perioperative mortality, and overall survival

During a median follow-up of 41.0 months (25th–75th: 18.1–74.7), 425 patients (40.2%) died: 146 (34.4%) were ICs and 279 (65.6%) were non-ICs.

Causes of death were tumor progression in 202 (47.5%), hepatic failure in 59 (13.9%), cardiovascular disease in 17 (4.0%), extrahepatic cancer in 18 (4.2%), hemorrhage in 13 (3.1%), infections in 11 (2.6%), embolism in 3 (0.7%), renal failure in 2 (0.5%), and other causes in 11 patients (2.6%). In 89 patients (20.9%), the cause of death was not reported. The overall distribution of causes of death did not differ among the 5 groups (Table 1). However, focusing on liver failure as a cause of death, hyperbilirubinemic+CSPH patients showed a significantly greater propensity to die from this cause compared to ICs.

The 1-month, 3-month, and 6-month mortality rates (perioperative mortality) of hyperbilirubinemic, CSPH, and mHCC groups did not significantly differ from those of ICs, whereas patients with hyperbilirubinemia+CSPH showed a significantly higher mortality at 3 and 6 months (*p*=0.043 and *p*<0.001, respectively) (Supplemental Table S1, http://links.lww.com/HC9/C67).

**FIGURE 3:**
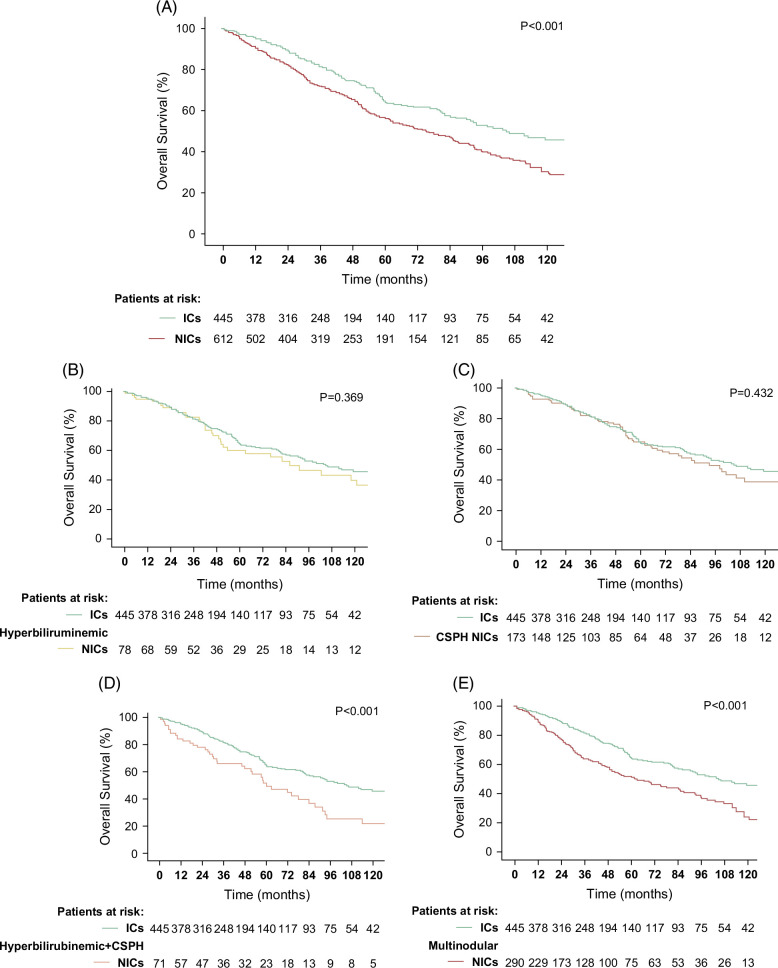
Overall survival in the whole population of resected patients (ideal candidates vs. non-ideal candidates) (A); in patients with isolated hyperbilirubinemia versus ideal candidates (B); in patients with CSPH versus ideal candidates (C); in patients with hyperbilirubinemia+CSPH versus ideal candidates (D); in patients with multinodular HCC versus ideal candidates (E). Abbreviations: CSPH, clinically significant portal hypertension; ICs, ideal candidates; NICs, non-ideal candidates.

The median OS was longer in ICs than in non-ICs (104.9 mo, 95% CI 78.9–130.9 vs. 75.3 mo, 95% CI 63.9–86.7; *p*<0.001) (Figure 3A). However, patients with isolated hyperbilirubinemia (86.0 mo, 95% CI 56.1–116.0; *p*=0.356) or CSPH (93.1 mo, 95% CI 74.1–112.1; *p*=0.432) showed a non-statistically different median OS compared to ICs (Figures 3B, C). Instead, median OS of patients with hyperbilirubinemia+CSPH (60.0 mo, 95% CI 42.6–77.4; *p*<0.001) or mHCC (ICs 61.9 mo, 95% CI 45.0–78.9; *p*<0.001) were significantly shorter compared to that of ICs (Figures 3D, E).

The 1-year, 3-year, and 5-year survival rates of the different subgroups were:

- ICs: 95.5%, 81.6%, and 65%;
- isolated hyperbilirubinemia: 94.7%, 82.8%, and 60.1%;
- isolated CSPH: 92.7%, 81.3%, and 64.8%;
- hyperbilirubinemia+CSPH: 84.1%, 66.0%, and 51.6%;
- mHCC: 89.6%, 64.0%, and 51.2%.

The analysis of OS across calendar periods revealed a difference between ICs and non-ICs, as survival of ICs significantly improved in the last period, while it did not change in non-ICs (Supplemental Figure S1, http://links.lww.com/HC9/C68).

## DISCUSSION

The appropriate use of HR to treat patients with HCC remains a controversial issue, being this decision guided by different conceptual positions,^1^^–^^4^^,^^28^^,^^29^ and different real-world approaches in the Western and Eastern world. The 2022 BCLC algorithm,^3^ a recognized therapeutic proposal for HCC, does not recommend HR as front-line therapy for non-transplantable patients with CSPH or hyperbilirubinemia or mHCC, considering encouraging but not probative the results of many studies (including one randomized trial^11^) reporting good outcomes of HR in patients with CSPH^6^^–^^8^^,^^10^ and its superiority over TACE and ablation in patients with mHCC.^9^^,^^12^^–^^19^ Nevertheless, a modest expansion of the gateway to surgery is present in the 2022 BCLC algorithm,^5^ as laparoscopic HR could be considered in patients with a *mild* degree of portal hypertension (although a cutoff is not indicated), while the position about hepatectomy for mHCC (limited to 3 nodules) remains pending waiting for the results of future, prospective clinical research, and HR is excluded for patients with intermediate or advanced HCC. Conversely, the multiparametric therapeutic hierarchy (MTH) approach^1^ and the recent European and Italian guidelines,^2^^,^^27^ assembled according to the Oxford Centre and GRADE methodology, respectively, suggest a broader use of HR. For these updated guidelines, both CSPH and Child–Pugh class B cirrhosis are not absolute contraindications for limited HR, but their adverse impact on the outcome should be weighed by MTB with a personalized approach, also considering the potential benefit over alternative treatments. As far as mHCC is concerned, both Korean and Italian guidelines are permissive for patients with oligo-nodular HCC (2–3 nodules),^26^^,^^27^ while Japanese and Asian guidelines accept surgery for multiple tumors.^28^^,^^29^ The present study yields relevant findings on this debated topic.

First, we found that, over the 23 years of the study period, the majority (around 60%) of resected patients in real-world had at least one clinical feature—modest hyperbilirubinemia, CSPH, or mHCC—that placed them into the non-IC group, and their percentage did not significantly change over time. Hence, despite the MTH strategy—suggesting to explore the feasibility of curative treatments before accepting palliative ones^1^—has been systematized only recently, the long-lasting predominance of non-ICs in our and previous surgical series^6^^–^^8^^,^^10^^,^^15^ indicates that its key concept has long been rooted in clinical practice.

However, we observed that non-ICs were a heterogeneous population, with a changing composition over time. The progressive increase in the prevalence of patients with mHCC and the opposite change for patients with hyperbilirubinemia and hyperbilirubinemia+CSPH suggests a tendency to expand the use of surgery along the line of tumor features, and a growing caution in the presence of hallmarks of liver dysfunction, even in Child–Pugh A patients.

As the second main result, we found room for a prudent expansion of the BCLC criteria for eligibility to HR without compromising its outcome. Namely, we excluded from the analysis patients with PS >2 or severe liver dysfunction (bilirubin >2 mg/dL), and only 20 patients had a platelet count (<50,000/mm^3^), indirectly suggesting the presence of a high-grade PH. Additionally, among mHCC patients, only 14.5% had >3 nodules. Using these selection criteria, isolated hyperbilirubinemia or CSPH did not significantly compromise the OS compared to ICs, suggesting that HR, if judged feasible, should be privileged over non-curative therapies in non-transplantable patients with these characteristics, to avoid the risk of undertreatment. However, it could be argued that the systematic adoption of the MTH principle might privilege the initial therapeutic results and not necessarily the long-term survival, which represents the actual goal. The intercontinental Bridge study,^10^ which had already shown that either isolated CSPH or hyperbilirubinemia has minimal impact on OS, did not solve this dilemma due to a short follow-up (27 mo). Moreover, a selection bias, caused by the refusal to submit data for the study by 22 out of 42 centers, could have affected its results. Instead, our conclusions rely on both perioperative and long-term OS, and they are supported by the fact that treatments of tumor recurrence did not differ between ICs and non-ICs. Therefore, our results corroborate, in European patients (who represented a minority of cases in the Bridge study) and with a longer follow-up, those obtained in that study.

Together with the degree of portal hypertension and liver dysfunction (measurable in several ways), the calculation of the HR extent to be radical is crucial for an appropriate selection of candidates,^2^^,^^3^^,^^23^^,^^26^^,^^31^ and a remnant liver volume >40% is recommended in patients with cirrhosis.^32^ This carefulness is therefore mandatory, particularly in non-ICs for liver dysfunction and/or CSPH, who can tolerate minor parenchymal sacrifice compared to ICs. In our series, major HRs were exceedingly rare, except for the mHCC group. Therefore, our results may be representative of what is achievable with limited resections. In addition, it is pertinent to note that, in patients with mHCC, HR was more likely to be adopted when nodules had a monolobar rather than a bilobar distribution, which increases the complexity of surgery.

The third main finding of our study is the demonstration that the co-presence of hyperbilirubinemia and CSPH or the presence of mHCC were detrimental to the outcome of HR, confirming previous reports.^6^^,^^10^^,^^33^^,^^34^ However, hepatectomy may still yield the best survival in most of these patients, who therefore represent the specific populations for which future head-to-head comparisons between HR and non-surgical therapies are warranted. In the meantime, guidance for therapeutic decisions may rely on the prognostic model (based on tumor diameter and number of nodules) developed by Kawaguchi et al^16^ on 43,904 patients who underwent HR, TACE, or ablation, and externally validated in an international cohort. The Appendix (Supplemental Material, http://links.lww.com/HC9/C67) reports the application of this score to some of our patients with hyperbilirubinemia+CSPH, or mHCC, showing that HR ordinarily outperforms ablation and TACE in terms of long-term survival.

Postoperative mortality, which in cirrhotic patients resected for HCC should be maintained <3%,^5^ remained below this threshold in all groups except for non-ICs with hyperbilirubinemia+CSPH, in whom it increased to 4.5% at 3 months and to 13.1% at 6 months, probably due to the more compromised liver function of these patients compared to ICs. In line with this assumption, patients with hyperbilirubinemia+CSPH showed the highest mortality rate from liver failure.

At partial variance, in the Bridge study^10^ non-ICs showed a higher short-term mortality than ICs but, in that study, the analysis included non-ICs taken together, a higher upper limit of bilirubin was accepted (7.8 mg/dL), and the HR results did not benefit from the improvements in surgical and postoperative procedures picked up by our more recent investigation.

Instead, the poorer OS of patients with mHCC can only be attributable to a dismal oncological history, as suggested by their highest propensity to develop early recurrences, as already reported,^35^^,^^36^ and the shortest TTNT.

### Shortcomings of the study

The retrospective nature of the study makes it vulnerable to unintended biases, and although the ITA.LI.CA database includes data of “all comers” (see the Methods section), a selection bias can only be excluded using data from nationwide compulsory registries. Moreover, although the 1-month postoperative mortality rate did not significantly change between groups, our study failed to capture a possible difference between ICs and non-ICs in non-fatal postoperative complications, such as liver decompensation and liver failure, as well as in the length of hospital stay, as pertinent data are not available in our database. In fact, any degree of liver dysfunction and portal hypertension increases the risk of a more complicated perioperative outcome.

More importantly, this study does not identify the upper limits of isolated hyperbilirubinemia or CSPH (for instance, using platelet count as a surrogate marker) beyond which the outcome of HR significantly declines with respect to ICs. However, considering that non-ICs had a certain—although not statistically significant—reduction in OS compared to ICs, it is conceivable that the raw measurements we chose approximate these limits.

Another drawback of our study is the lack of pre-defined uniform surgical techniques and postoperative assistance used across centers, which represent unavoidable shortcomings of real-world, retrospective, and multicentric investigations.

Moreover, we missed the modality of HR (laparoscopic or laparotomic) as this information is lacking in the ITA.LI.CA database. Since mini-invasive surgery has been proven to improve HR outcome and can expand the use of surgery to more fragile patients,^37^^,^^38^ our real-world results should be retested when laparoscopic/robotic surgery has fully replaced open surgery.

Lastly, among non-ICs, a comparison of outcomes between resected patients and those treated according to the BCLC indications would further refine the management of HCC patients. This comparison will be the subject of an additional analysis of our database.

### Strengths of the study

Our results have been generated from a large sample size and over a long time period allowing us to describe the evolutionary scenario of HR. Moreover, the long follow-up provided solid information on long-term outcome of HR in different categories of patients.

Despite the current Western recommendations to assess the presence and degree of portal hypertension by measuring the porto-hepatic venous pressure gradient,^39^^,^^40^ most centers do not routinely perform this measurement but rely on imaging (presence of esophageal varices, dilated portal trunk, splenomegaly) and low platelet count to detect portal hypertension. Therefore, our study, adopting these methods, provides more usable reference points for HR.

Finally, our findings provide a benchmark for testing the potential benefit of adjuvant immunotherapy in different subgroups of resected patients.

## Supplementary Material
