# Supplementary material for: Outcome of hepatic resection for HCC in ideal and non-ideal candidates
Source: Hepatol Commun. 2025 Jul 29;9(8):e0772. doi: 10.1097/HC9.0000000000000772 (PMC12306702; doi:10.1097/HC9.0000000000000772)
Supplement: Supplementary file 1 [file hc9-9-e0772-s001.docx]

**Supplementary Table 1.** One, three- and six-month mortality rate in the subsets of resected patients.

|  | **1-month mortality rate** | **3-month mortality rate** | **6-month mortality rate** | **p-values ^1^** | **p-values ^2^** | **p-values ^3^** |
| --- | --- | --- | --- | --- | --- | --- |
| **Ideal candidates, N=445** | 0.2% | 1.2% | 2.7% | - | - | - |
| **Hyperbilirubinemia, N=78** | 1.3% | 1.3% | 5.6% | 0.163 | 0.913 | 0.185 |
| **CSPH, N=173** | 0.6% | 1.1% | 3.8% | 0.487 | 0.976 | 0.482 |
| **Hyperbilirubinemia+CSPH, N=71** | 1.4% | 4.5% | 13.1% | 0.136 | **0.043** | **<0.001** |
| **mHCC, N=290** | 0.3% | 2.1% | 3.8% | 0.760 | 0.291 | 0.407 |

**Abbreviations:** CSPH, clinically significant porta hypertension; mHCC, multinodular hepatocellular carcinoma.

^1^: 1-month mortality compared to ideal candidates; ^2^: 3-month mortality compared to ideal candidates; ^3^: 6-month mortality compared to ideal candidates.

**Appendix.** Survival rates at 1, 3-and 5 years of 10 patients with hyperbilirubinemia+CSPH or multinodular HCC calculated according to the prognostic model developed by Kawaguchi et al. [26]. The link for the access to the calculator is: <http://www.u-tokyo-hbp-transplant-surgery.jp/about/calculation.html>

| **Patient** | **Number of nodules** | **Diameter of the biggest nodules (cm)** | **Age range at the time of HR (years)** | **Bilirubin (mg/dL)** | **Platelet count**  **(*10^3^/µl)** | **Albumin (g/dL)** | **Patient group** | **Treatment** | **Survival rates (calculated)^a^** | | |
| --- | --- | --- | --- | --- | --- | --- | --- | --- | --- | --- | --- |
|  |  |  |  |  |  |  |  |  | **1 years** | **3 years** | **5 years** |
| 001 | 2 | 6 | 61-73 | 0.6 | 180 | 3.6 | NIC: multinodular, normal bilirubin and absence of CSPH | HR | 86.0% | 58.2% | 38.6% |
|  |  |  |  |  |  |  |  | TACE | 85.4% | 48.2% | 25.0% |
|  |  |  |  |  |  |  |  | Ablation | NA | NA | NA |
| 002 | 4 | 2.5 | 61-73 | 0.88 | 204 | 3.3 | NIC: multinodular, normal bilirubin and absence of CSPH | HR | 89.8% | 68.0% | 50.7% |
|  |  |  |  |  |  |  |  | TACE | 87.5% | 54.0% | 31.0% |
|  |  |  |  |  |  |  |  | Ablation | 90.6% | 60.0% | 36.0% |
| 003 | 2 | 1.8 | 61-73 | 0.9 | 88 | 3.3 | NIC: multinodular, normal bilirubin and presence of CSPH | HR | 93.2% | 77.5% | 63.8% |
|  |  |  |  |  |  |  |  | TACE | 91.3% | 65.8% | 45.2% |
|  |  |  |  |  |  |  |  | Ablation | 94.1% | 73.3% | 53.4% |
| 004 | 2 | 1.5 | 61-73 | 0.5 | 150 | 3.6 | NIC: multinodular, normal bilirubin and presence of CSPH | HR | 94.1% | 80.2% | 67.9% |
|  |  |  |  |  |  |  |  | TACE | 92.2% | 68.6% | 48.9% |
|  |  |  |  |  |  |  |  | Ablation | 95.5% | 79.1% | 62.3% |
| 005 | 2 | 2.5 | 61-73 | 1.3 | 115 | 3.7 | NIC: multinodular, hyperbilirubin, absence of CSPH | HR | 92.2% | 74.5% | 59.6% |
|  |  |  |  |  |  |  |  | TACE | 90.5% | 63.0% | 41.6% |
|  |  |  |  |  |  |  |  | Ablation | 93.1% | 69.6% | 48.0% |
| 006 | 5 | 3 | 61-73 | 1.2 | 127 | 3.4 | NIC: multinodular, hyperbilirubin, absence of CSPH | HR | 87.2% | 61.1% | 42.0% |
|  |  |  |  |  |  |  |  | TACE | 85.5% | 48.4% | 25.2% |
|  |  |  |  |  |  |  |  | Ablation | 88.2% | 52.8% | 27.5% |
| 007 | 3 | 3 | 61-73 | 1.2 | 70 | 2.9 | NIC: multinodular, hyperbilirubin and presence of CSPH | HR | 89.7% | 67.6% | 50.3% |
|  |  |  |  |  |  |  |  | TACE | 87.3% | 53.4% | 30.4% |
|  |  |  |  |  |  |  |  | Ablation | 91.4% | 63.1% | 39.4% |
| 008 | 2 | 4.5 | 61-73 | 1.2 | 180 | 3.7 | NIC: multinodular, hyperbilirubin and presence of CSPH | HR | 88.2% | 63.5% | 45.0% |
|  |  |  |  |  |  |  |  | TACE | 87.2% | 53.1% | 30.1% |
|  |  |  |  |  |  |  |  | Ablation | NA | NA | NA |
| 009 | 1 | 7 | 57-72 | 1.6 | 71 | 4 | NIC: single nodule, hyperbilirubin and CSPH | HR | 90.1% | 68.8% | 51.8% |
|  |  |  |  |  |  |  |  | TACE | 87.9% | 55.1% | 32.3% |
|  |  |  |  |  |  |  |  | Ablation | NA | NA | NA |
| 010 | 1 | 3 | 57-72 | 1.23 | 96 | 3.8 | NIC: single nodule, hyperbilirubin and CSPH | HR | 93.1% | 77.3% | 63.6% |
|  |  |  |  |  |  |  |  | TACE | 92.3% | 69.0% | 49.4% |
|  |  |  |  |  |  |  |  | Ablation | 94.4% | 74.7% | 55.4% |

**Abbreviations:** CSPH, clinically significant portal hypertension; HCC, hepatocellular carcinoma; HR, hepatic resection; NA not available (because nodule size >3 cm); NIC, non-Ideal candidate; TACE, transarterial chemoembolization.
