# Supplementary material for: Outcome of hepatic resection for HCC in ideal and non-ideal candidates
Source: Hepatol Commun. 2025 Jul 29;9(8):e0772. doi: 10.1097/HC9.0000000000000772 (PMC12306702; doi:10.1097/HC9.0000000000000772)

Supplementary Figure 1. Overall survival across calendar periods of ideal candidates (A) and non-ideal candidates (B).

Supplementary Figure 1

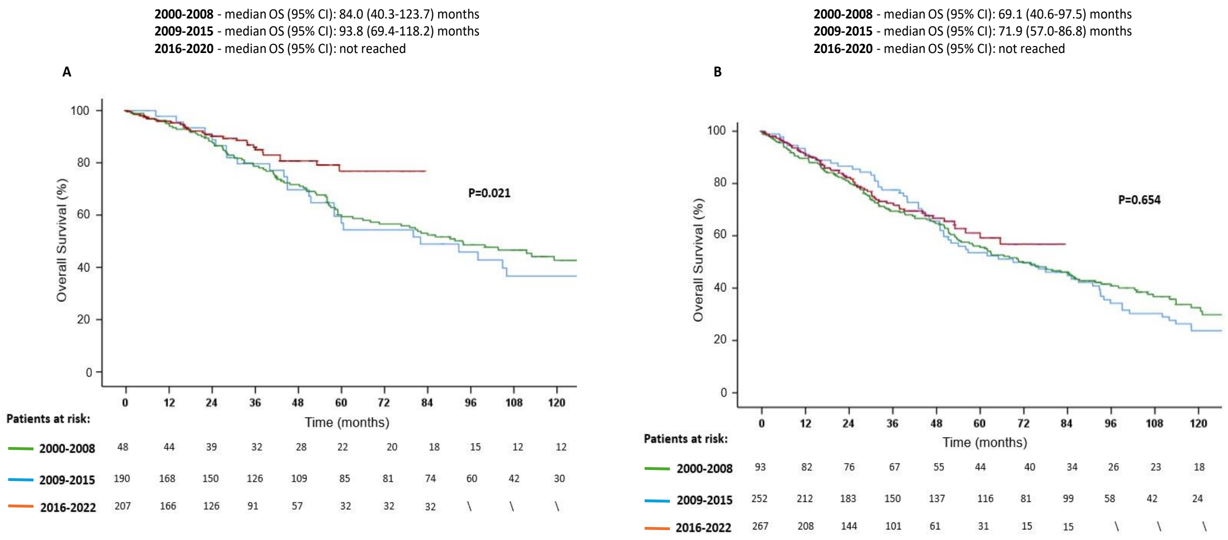

Supplement: Supplementary file 2 [file hc9-9-e0772-s002.pdf]
